# Supplementary material for: Structure of human steroid 5α-reductase 2 with the anti-androgen drug finasteride
Source: Nat Commun. 2020 Oct 27;11:5430. doi: 10.1038/s41467-020-19249-z (PMC7591894; doi:10.1038/s41467-020-19249-z)
Supplement: Supplementary file 1 — Supplementary Information [file 41467_2020_19249_MOESM1_ESM.pdf]

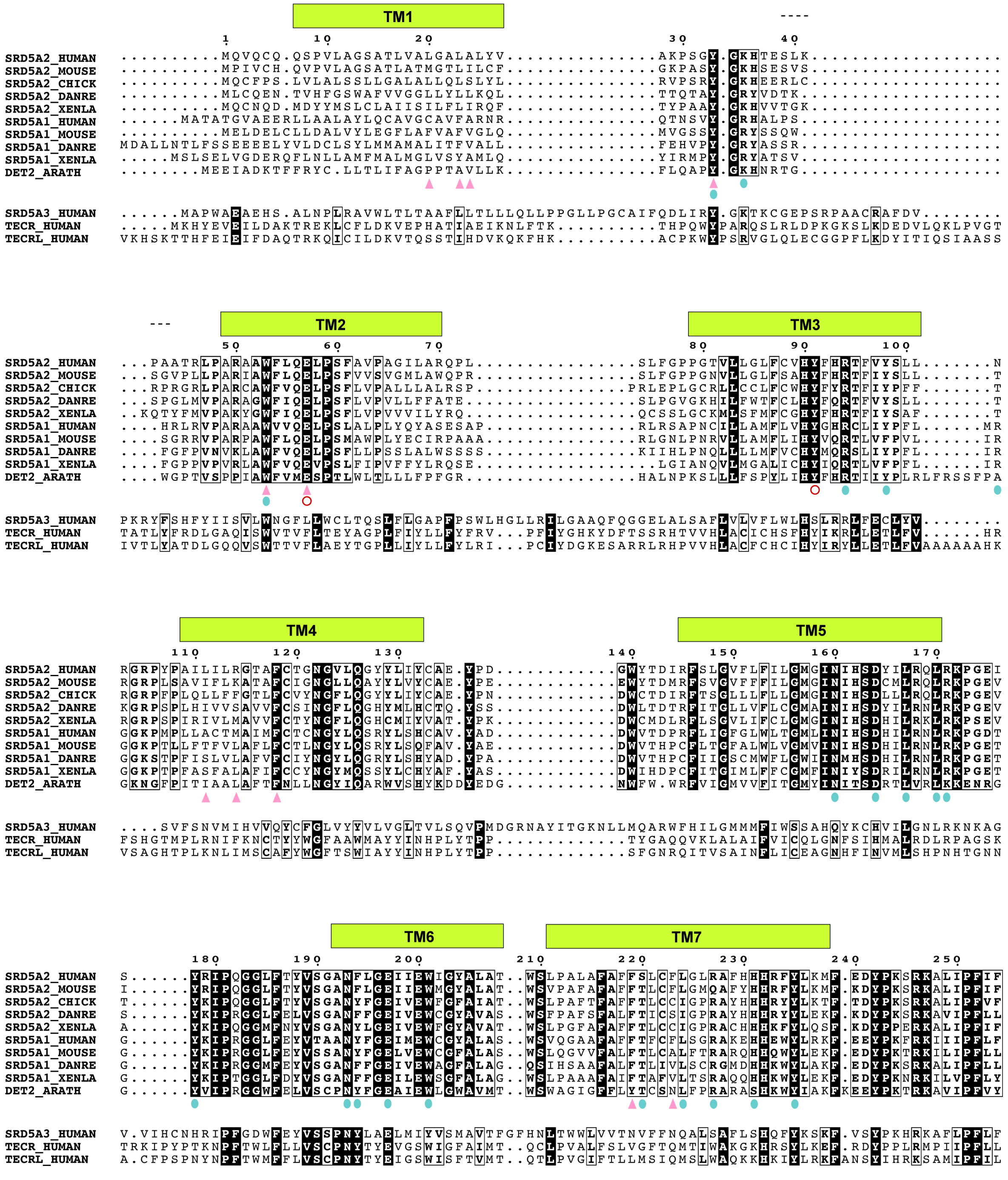

Extended Data Figure 1. Multi-sequence alignments of SRD5A enzymes.

The sequences of the SRD5A1/2 subfamily members across different species from human to plant were aligned first. The other SRD5A family members in human containing SRD5A3, GSPN2 (TECR), and GSPN2-like (TECRL) were then aligned to the first alignment. The species were represented by "DANRE" for *Danio rerio* and "XENLA" for *Xenopus laevis*. DET2 is the SRD5A1/2 homologue in *Arabidopsis thaliana*. The transmembrane helical regions are labeled

above the alignment. Residues that are identical and highly similar in each alignment are shown in black and white boxes, respectively. The residues involved in the DHF-binding and NADP-binding are indicated by pink triangles and cyan circles, respectively. The two residues involved in the catalysis are indicated by open circles.

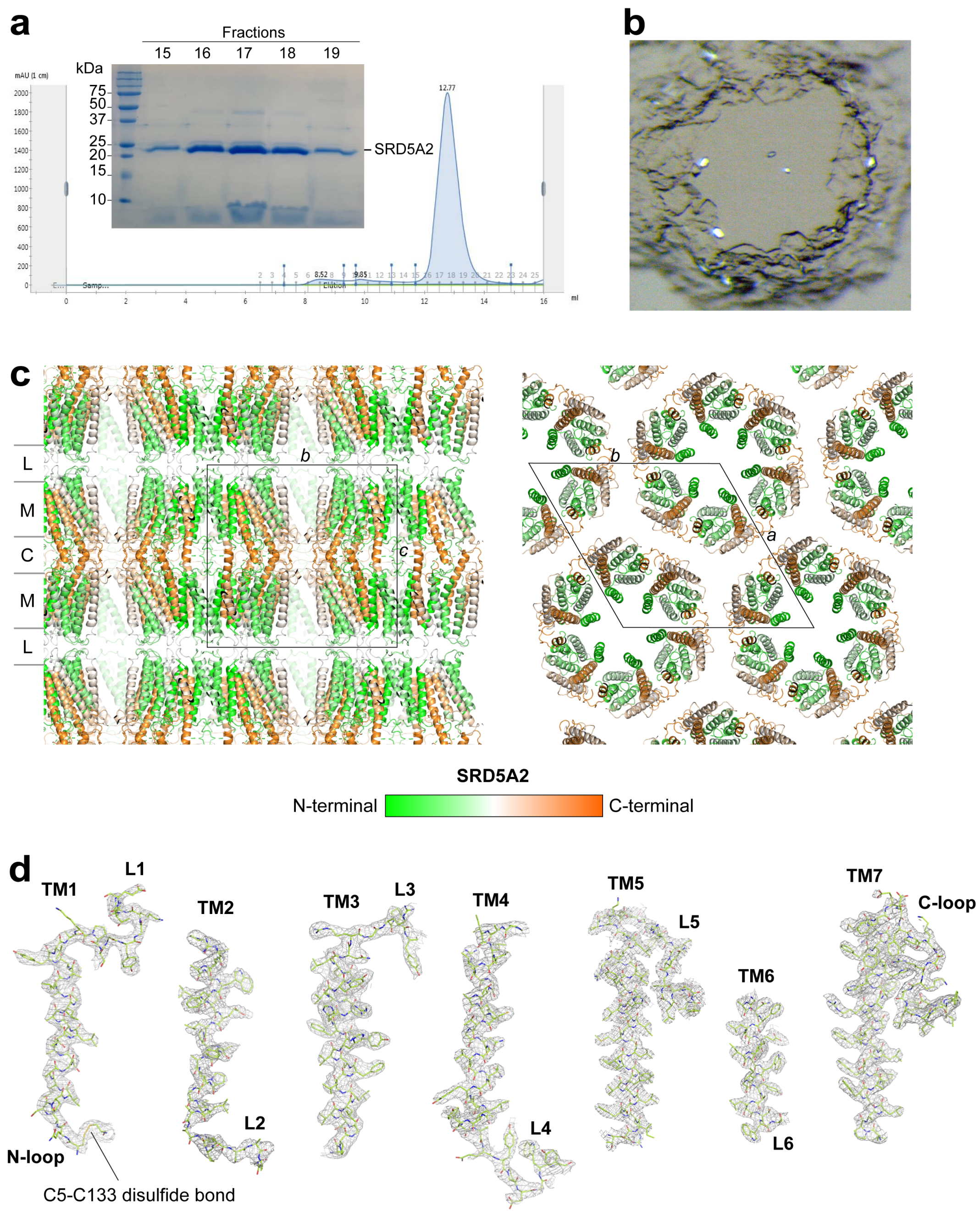

Extended Data Figure 2. SRD5A2 purification and crystallization.

(a) Purification of SRD5A2 by size-exclusion chromatography (SEC). The protein homogeneity was high as indicated by the monodispersed peak in SEC and the following SDS-PAGE analysis. **(b)** Crystals of SRD5A2 in the lipid mesophase. (**c**) Two views of crystal packings in the SRD5A2 crystal. A unit cell was indicated. It is interesting to note the *P622* space group of the SRD5A2 crystals is very uncommon for membrane protein crystals from the lipid mesophase. SRD5A2 forms crystallographic hexamers on each lipid bilayer. The cytosolic region, transmembrane

region, and lumenal region of SRD5A2 in the crystal packings are indicated as “C”, “M”, and “L”, respectively. (**d**) *2Fo-Fc* electron density maps of each transmembrane helix. The map of the C5-C133 disulfide bond is also shown.

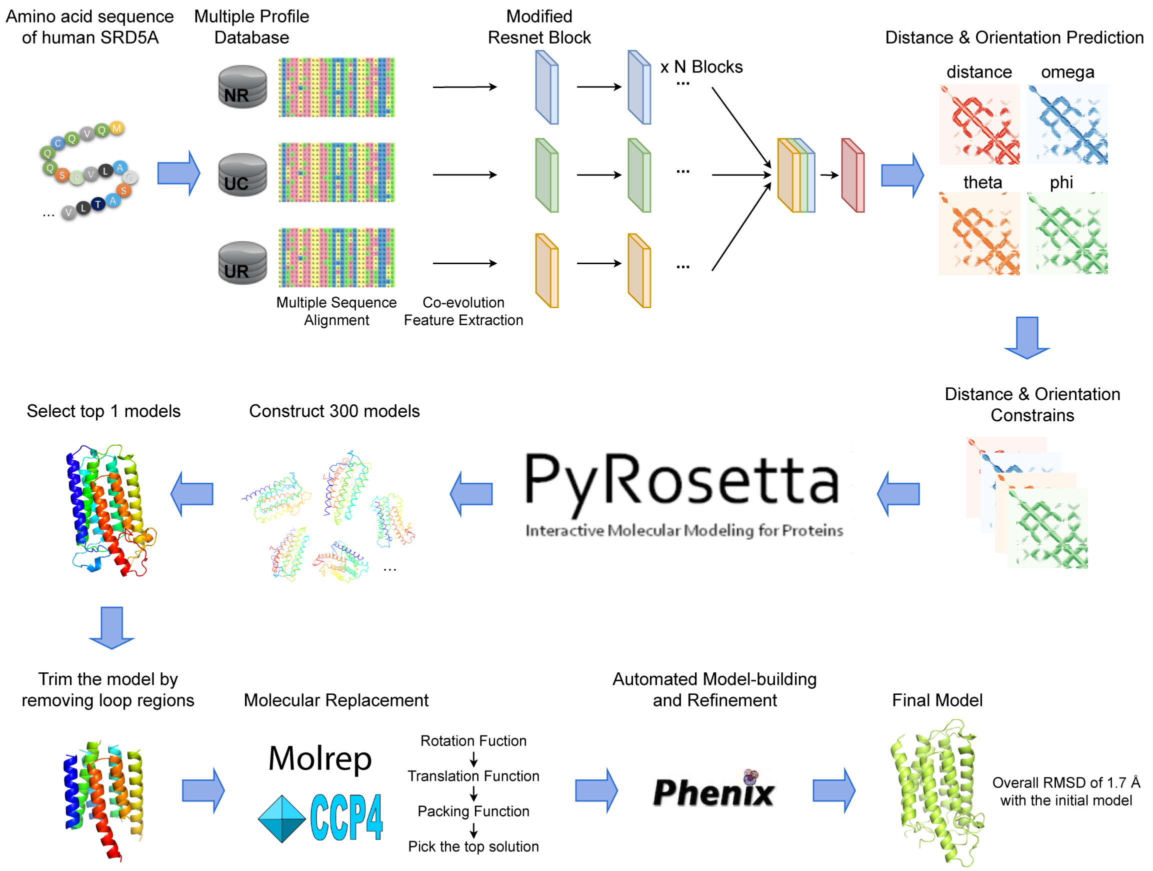

Extended Data Figure 3. *de novo* prediction of SRD5A2 structure and molecular replacement. We constructed the model using a *de novo* approach from a predicted distance/orientation matrix, which was derived from a variety of multiple sequence alignments (MSAs) by a multi-branch fusion ResNet. Following the trRosetta methodology, we generated 300 models from the predicted distances and orientations using constrained minimization, which is an embedded module from PyRosetta. Finally, the top 1 model with the lowest Rosetta energy was selected as the input model for molecular replacement (MR) to determine the protein structure. All references for the software are cited in the Methods section.

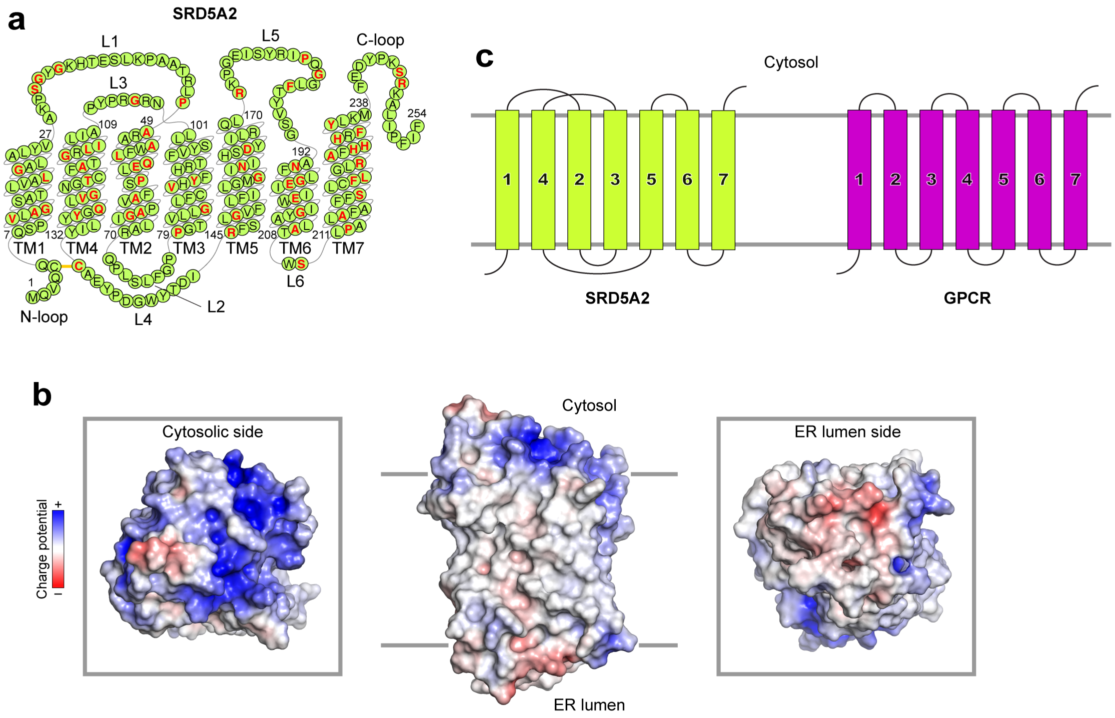

Extended Data Figure 4. Topological analysis of SRD5A2.

(a) The amino acid sequence of SRD5A2 in different TM and loop regions. The disease-associated missense/nonsense mutation sites were colored in red. The C5-C133 disulfide bridge was indicated by a connecting line between the two cysteine residues. **(b)** Surface charge potential analysis of SRD5A2. Positively charged regions indicates the cytosolic side. **(c)** The different topological arrangement of the seven TMs in SRD5A2 and GPCRs.

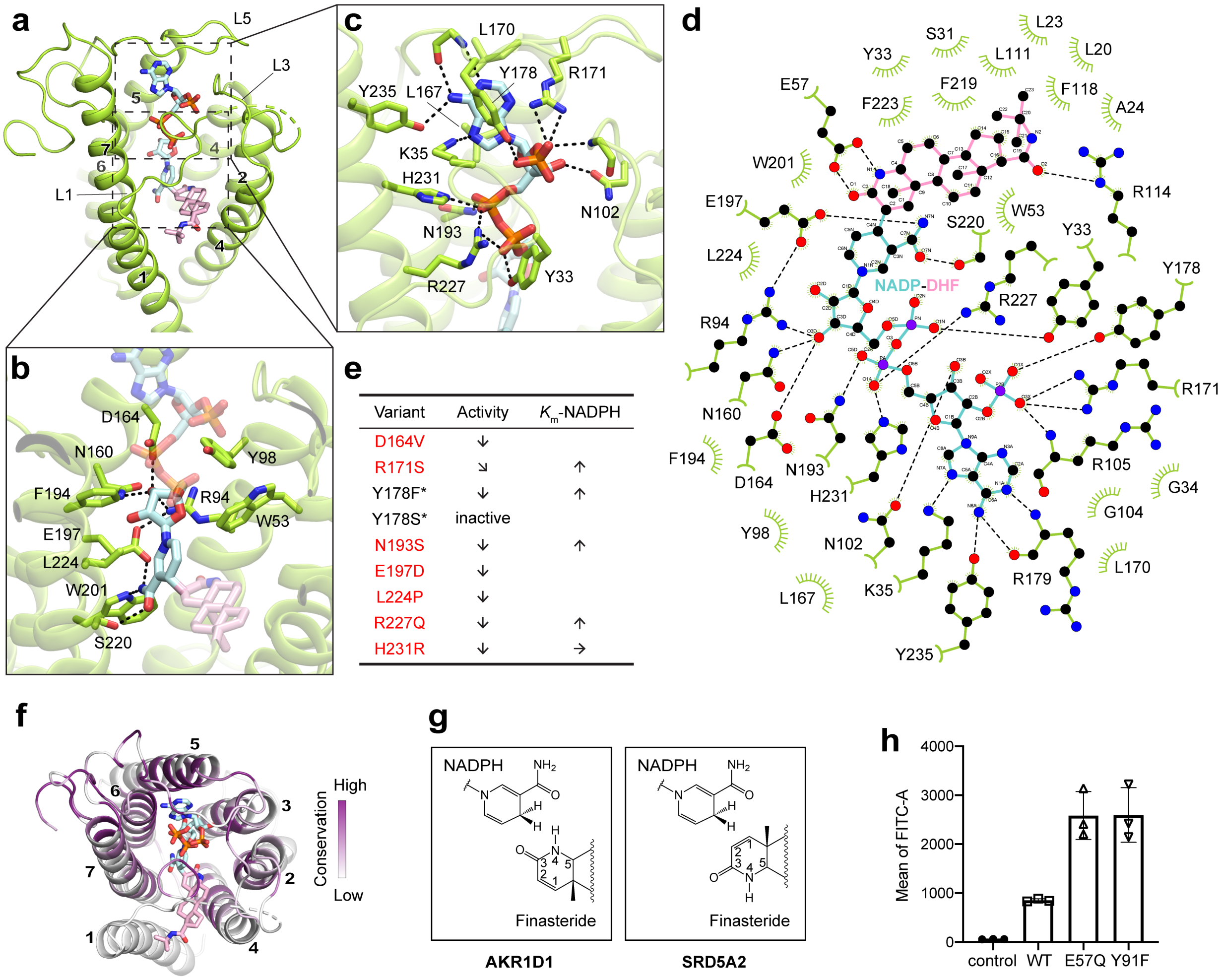

Extended Data Figure 5. Molecular details of ligand-binding cavity, finasteride inhibition and mutagenesis data.

(a) The overall view of the NADP-binding pocket. **(b)** The detailed interactions between the nicotinamide-ribose moiety of NADP and SRD5A2. **(c)** The detailed interactions between the diphosphate moiety of NADP and SRD5A2. **(d)** The NADP-DHF/SRD5A2 interaction analysis. The polar and hydrophobic interactions were shown as a 2D view prepared by LigPlot^{+} (https://www.ebi.ac.uk/thornton-srv/software/LigPlus/). **(e)** Summary of previous enzymologically studied mutations in the NADP-binding interface. The mutations found in patients with steroid 5α-reductase deficiency were colored in red. The disruptive effects of the Y178F and Y178S mutations were derived from the mutagenesis study of rat SRD5A2. **(f)** Sequence conservation analysis on SRD5A1 and SRD5A2. The conservation is derived from the sequence alignment of SRD5A1 and SRD5A2 as shown in Figure S1. **(g)** Different orientations of finasteride relative to NADPH in AKR1D1 and SRD5A2 leading to different inhibition mechanisms. (**h**) Cell surface expression levels of SRD5A2 and its two mutants. The membrane fractions from these cells were used for measuring enzymatic activities as shown in Fig. 3e.

Expression levels were determined by fluorescent antibody staining of Sf9 cells. All data are presented as mean ± SEM of 3-4 independent experiments.

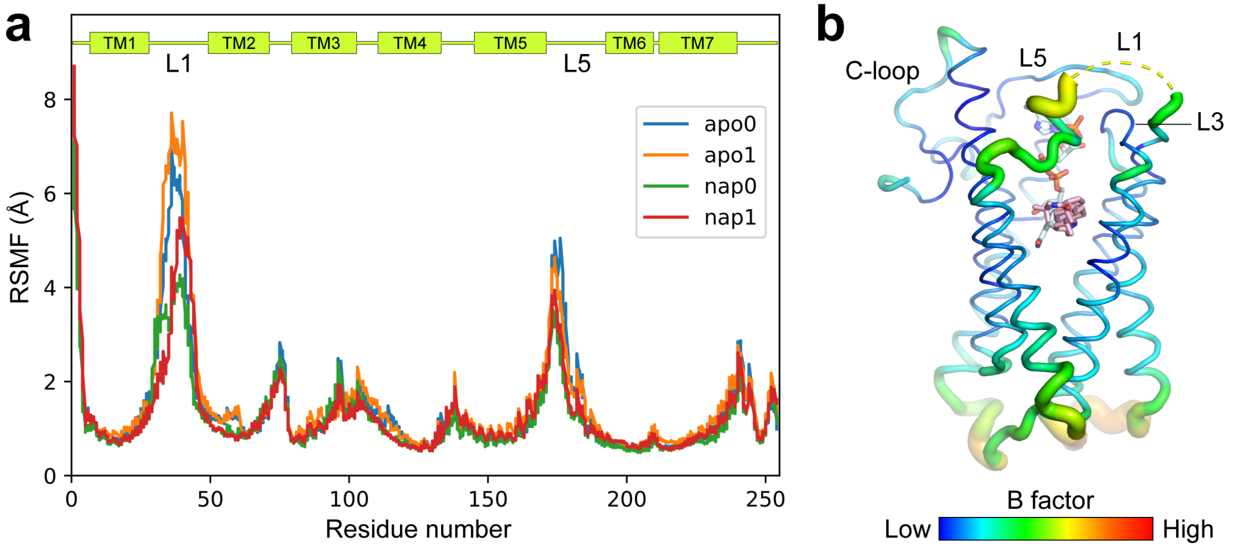

Extended Data Figure 6. The dynamic feature of the cytosolic loops in SRD5A2.

(a) Root-Mean-Square-Fluctuation (RMSF) plots of the protein calculated from the two apo and two nap simulation states. The cytosolic loops, L1 and L5 show the largest fluctuation in simulation. (**b)** The B-factor putty view of the SRD5A2 structure showing the relatively higher B-factors in the L1 region.
