# Supplementary material for: Structure of human steroid 5α-reductase 2 with the anti-androgen drug finasteride
Source: Nat Commun. 2020 Oct 27;11:5430. doi: 10.1038/s41467-020-19249-z (PMC7591894; doi:10.1038/s41467-020-19249-z)
Supplement: Supplementary file 5 — Reporting Summary [file 41467_2020_19249_MOESM5_ESM.pdf]

nature research

Corresponding author(s): Hao Fan, Zhiyi Wei, Cheng Zhang

Last updated by author(s): Sep 23, 2020

Reporting Summary

Nature Research wishes to improve the reproducibility of the work that we publish. This form provides structure for consistency and transparency in reporting. For further information on Nature Research policies, see our Editorial Policies and the Editorial Policy Checklist.

Statistics

For all statistical analyses, confirm that the following items are present in the figure legend, table legend, main text, or Methods section.

n/a	Confirmed	
☐	☒	The exact sample size (*n*) for each experimental group/condition, given as a discrete number and unit of measurement
☐	☒	A statement on whether measurements were taken from distinct samples or whether the same sample was measured repeatedly
☒	☐	The statistical test(s) used AND whether they are one- or two-sided<br>*Only common tests should be described solely by name; describe more complex techniques in the Methods section.*
☒	☐	A description of all covariates tested
☒	☐	A description of any assumptions or corrections, such as tests of normality and adjustment for multiple comparisons
☐	☒	A full description of the statistical parameters including central tendency (e.g. means) or other basic estimates (e.g. regression coefficient) AND variation (e.g. standard deviation) or associated estimates of uncertainty (e.g. confidence intervals)
☒	☐	For null hypothesis testing, the test statistic (e.g. *F*, *t*, *r*) with confidence intervals, effect sizes, degrees of freedom and *P* value noted<br>*Give P values as exact values whenever suitable.*
☒	☐	For Bayesian analysis, information on the choice of priors and Markov chain Monte Carlo settings
☒	☐	For hierarchical and complex designs, identification of the appropriate level for tests and full reporting of outcomes
☒	☐	Estimates of effect sizes (e.g. Cohen's *d*, Pearson's *r*), indicating how they were calculated

Our web collection on statistics for biologists contains articles on many of the points above.

Software and code

Policy information about availability of computer code

Data collection	JBluIce
Data analysis	HKL2000, Coot, Phenix, CCP4, PyMol, MolProbity, trRosetta, Glide, Modeller, CHAMM-GUI, Visual Molecular Dynamics, APBS, NAMD 2.13, pyPcazip, Prism 8, NAMD.

For manuscripts utilizing custom algorithms or software that are central to the research but not yet described in published literature, software must be made available to editors and reviewers. We strongly encourage code deposition in a community repository (e.g. GitHub). See the Nature Research guidelines for submitting code & software for further information.

Data

Policy information about availability of data

All manuscripts must include a data availability statement. This statement should provide the following information, where applicable:

- Accession codes, unique identifiers, or web links for publicly available datasets
- A list of figures that have associated raw data
- A description of any restrictions on data availability

The coordinates and structure factors have been deposited in the Protein Data Bank under the accession codes PDB 7BW1. Distribution of research materials generated in this study including plasmids will need appropriate Material Transfer Agreements (MTAs). PDB IDs: 4QUV and 5V7P have been referenced in the manuscript. DOI link for each PDB code has been provided.

Field-specific reporting

Please select the one below that is the best fit for your research. If you are not sure, read the appropriate sections before making your selection.

☒ Life sciences ☐ Behavioural & social sciences ☐ Ecological, evolutionary & environmental sciences

For a reference copy of the document with all sections, see nature.com/documents/nr-reporting-summary-flat.pdf

Life sciences study design

All studies must disclose on these points even when the disclosure is negative.

Sample size	We performed mass spectrometry experiments. The method is robust, reliable and sensitive. For each data point, we repeated the experiment at least 3 times, which ensured a two-thirds (66%) probability that the averaged results were more accurate than a single experiment and allowed graphing and using statistical descriptions to evaluate our data.
Data exclusions	No.
Replication	All data points in Figure 3e were repeated 3-5 times independently. All attempts at replication were successful.
Randomization	No sample selection process was involved.
Blinding	Investigators were not blinded during experiments.

Reporting for specific materials, systems and methods

We require information from authors about some types of materials, experimental systems and methods used in many studies. Here, indicate whether each material, system or method listed is relevant to your study. If you are not sure if a list item applies to your research, read the appropriate section before selecting a response.

Materials & experimental systems

n/a	Involved in the study	
☐	☒	Antibodies
☐	☒	Eukaryotic cell lines
☒	☐	Palaeontology and archaeology
☒	☐	Animals and other organisms
☒	☐	Human research participants
☒	☐	Clinical data
☒	☐	Dual use research of concern

Methods

n/a	Involved in the study	
☒	☐	ChIP-seq
☒	☐	Flow cytometry
☒	☐	MRI-based neuroimaging

Antibodies

Antibodies used	Anti-FLAG M1 antibody produced by the authors.
Validation	The antibody has been validated in house by western blot.

Eukaryotic cell lines

Policy information about cell lines

Cell line source(s)	Insect Sf9 cells from Expression Systems
Authentication	The cell line has not been authenticated. We rely on the vendor for authentication. No cell type-specific experiments were performed.
Mycoplasma contamination	The cell line has not been tested for mycoplasma contamination.
Commonly misidentified lines (See ICLAC register)	None.
